# Prevalence of Gastroesophageal Reflux Disease (GERD) Among Electronic Cigarette-Smoking University Students in Jeddah, Saudi Arabia

**DOI:** 10.7759/cureus.35890

**Published:** 2023-03-08

**Authors:** Nouf A Alturki, Rahaf A Alghamdi, Raghad G Almehmadi, Rafeef M Derar, Roba M Waznah, Rose A Niyazi, Ghaidaa S Hasrat, Fayza F Alfayez, Ayman Elsamanoudy

**Affiliations:** 1 Department of Medicine, King Abdulaziz University Faculty of Medicine, Jeddah, SAU; 2 Department of Clinical Biochemistry, King Abdulaziz University Faculty of Medicine, Jeddah, SAU

**Keywords:** saudi arabia, jeddah, university students, smoking, e-cigarettes, gastroesophageal reflux disease (gerd)

## Abstract

Background

Gastroesophageal reflux disease (GERD) is one of the most common gastrointestinal tract diseases. Although there is a strong association between smoking and GERD, it is poorly understood until now. Electronic cigarettes (E-cigarettes) are widely used nowadays. So, our study aimed to investigate the prevalence of GERD among all Jeddah university students and its relation to E-cigarette smoking.

Methodology

A cross-sectional study was conducted among university students of all specialties in Jeddah, Saudi Arabia, using an online questionnaire to collect data distributed in a Google Form (Google LLC, Mountain View, California, United States) from August to November 2022.

Results

This study included 397 students, 36.5% of whom were from 18 to 20 years old, and the majority were females (69.3%). Of the participants, 43.8% were non-smokers, 13.1% were ex-smokers, and 43.1% currently smoked; of the last, 13.6% smoked tobacco cigarettes, 17.6% smoked hookah, and 35% were current E-cigarette smokers. The study found that among the participants, 19.9% had GERD based on the GerdQ, with females having a significantly higher percentage of GERD. A weak association was found between the prevalence of GERD and smoking cigarettes (p=0.49), hookah (p=0.988 ), and E-cigarettes (p=0.788 ) but this could be attributed to the high BMI.

Conclusion

E-cigarette smoking is more prevalent among university students in Jeddah than traditional cigarettes or hookah. However, there was no statistically significant link between E-cigarette smoking and GERD. High BMI could be a superadded factor.

## Introduction

One of the most prevalent digestive conditions is gastroesophageal reflux disease (GERD), in which stomach contents flow back into the esophagus and cause uncomfortable symptoms like heartburn, regurgitation, and mucosal damage-related symptoms [1].

The incidence of GERD is exceptionally high in Asian and Arab nations [2]. GERD significantly affects a patient's quality of life [3]. Furthermore, persistent acid reflux could lead to Barrett's esophagus, eventually leading to esophageal cancer, by irritating its mucosa [4].

Smoking and GERD have a considerable correlation, although the pathophysiologic link underlying this correlation is complicated and poorly understood [5,6]. Additionally, nicotine, which has the propensity to relax the smooth muscle of the lower esophageal sphincter, is thought to be the primary etiological cause [7]. Smoking can also trigger the stomach to produce more acid and intensify acidic secretions, which could cause additional esophageal injury [7]. As an alternative to traditional cigarettes that provides nicotine in the form of an aerosol without tobacco use, the vape or electronic cigarette (E-cigarette) has been marketed as a healthier option as proposed by the young generation [8].

Vapes include hazardous chemicals in a fluid mixture with nicotine, vegetable glycerin, propylene glycol, and various flavors [9]. E-cigarettes heat these compounds to create an aerosol [10]. Due to the lack of knowledge about the health effects of E-cigarettes and advertisements that portray E-cigarettes as preferable to traditional smoking, the use of the vape among teens and young people has recently grown in popularity in Saudi Arabia and worldwide [11].

An Al-Qassim University study that investigated the use of E-cigarettes among medical students found that around one in 10 admitted to using them [1]. For dental, pharmacy, and medical science students in Saudi Arabia, it is projected that smoking prevalence ranges from 7.9% to 13.4% to 29%, respectively. [11]. To the best of our knowledge, no thorough research has been done on the prevalence of GERD among university students who smoke E-cigarettes and the difference between this prevalence from other types of smoking in Jeddah city (the largest city in the western area of Saudi Arabia). So, this study aimed to assess the prevalence of GERD among university students in Jeddah and its relation to E-cigarette smoking.

## Materials and methods

Study design

A cross-sectional non-interventional study was done in Jeddah, Saudi Arabia, from August to November 2022. Ethical approval for the study was obtained from the Research Ethics committee of King Abdulaziz University, Faculty of Medicine, Jeddah, Saudi Arabia (approval number: 463-22).

Study participants

The inclusion criteria were smokers, former smokers, and non-smoker students from various specialties from all universities in Jeddah aged 18 and up, and the exclusion criteria were graduates and students who live outside of Jeddah.

Sample size

Using a margin of error of 5% and a confidence interval of 95%, a sample of 360 students was calculated using the online Raosoft sample size calculator [12].

Data collection

An online questionnaire was created with Google Forms (Google LLC, Mountain View, California, United States) and distributed through social media platforms including WhatsApp (WhatsApp LLC, Menlo Park, California, United States), Twitter (Twitter, Inc., San Francisco, California, United States), Telegram (Telegram Group Inc., Dubai, United Arab Emirates), and Snapchat (Snap Inc., Santa Monica, California, United States). Thefirst section of the questionnaire included items about the participants' demographics (gender, marital status, age, university, specialty, and college year) and the underlying medical history. The second section included items that are used to assess the smoking pattern (non-smokers, ex-smokers, or smokers (cigarettes, hookah, E-cigarettes). Moreover, it assessed the following questions: if they smoke tobacco, how many cigarettes, for how long they have been smoking, history of tobacco smoking, if they smoke hookah, and for how long, history of hookah smoking. Besides, if they ever used E-cigarettes, how many days per week, for how long, and for what reasons did they start (cheaper, variability of flavors, plans for reducing or quitting tobacco, decreasing smoking exposure to their loved ones, or avoiding public ban), and how long does the cartridge last?

The Gastroesophageal Reflux Disease Questionnaire (GerdQ) was used to assess GERD prevalence [13]. The GerdQ was validated as a subject-centered self-assessment through analysis of responses from qualitative interviews of more than 50 subjects to create a new six-item tool to asses the diagnosis and management of GERD (premonitory pilot testing). Then, subsequent revising of the questionnaire result was followed by a double-blind evaluation by two senior experts from the Family Medicine and Clinical Biochemistry departments. Those with a score of 8 or more were considered to have GERD, while those with a score of less than eight were not. [13,14].

Statistical analysis

Data were statistically analyzed using IBM SPSS Statistics for Windows, Version 26.0 (Released 2019; IBM Corp., Armonk, New York, United States). The Chi-squared test (χ2) was used for quantitative data expressed as numbers and percentages to assess the association between the variables. The association between the quantitative non-parametric variables expressed as mean and standard deviation (Mean ± SD) was examined using the Mann-Whitney test. Statistical significance was defined as a p-value of less than 0.05.

## Results

A total of 397 students were included in the study (122 males and 275 females). The data given in Table 1 shows that 45.6% of the students were 21-23 years old, 69.3% were females, and only 6.5% were married. The mean BMI was 22.78 ± 5.09kg/m^2^. Most students (76.8%) were from King Abdulaziz University, and 22.9% were in the fourth academic year. Of them, 11.3% had underlying medical problems, with asthma being the most common (40%).

**Table 1:** Distribution of studied students according to their demographics, BMI, academic data, and underlying medical problems (n=397) BMI, Body Mass Index; GERD, Gastroesophageal Reflux Disease; DM, Diabetes Mellites; HTN, Hypertension; IBS, Irritable Bowel Syndrome

Variable		n (%)
Age	18-20	145 (36.5)
	21-23	181 (45.6)
	24-26	61 (15.4)
	Older than 26	10 (2.5)
Gender	Female	275 (69.3)
	Male	122 (30.7)
Marital status	Married	26 (6.5)
	Single	371 (93.5)
BMI		22.78 ± 5.09
University	Al Ghad International College	2 (0.5)
	Batterjee Medical College	14 (3.5)
	Dar Alhekma University	3 (0.8)
	Effat University	2 (0.5)
	Fakeeh College	2 (0.5)
	Ibn Sina National College	10 (2.5)
	Jeddah University	51 (12.8)
	King Abdulaziz University	305 (76.8)
	King Saud bin Abdulaziz University	8 (2)
College year	1^st^	85 (21.4)
	2^nd^	71 (17.9)
	3^rd^	34 (8.6)
	4^th^	91 (22.9)
	5^th^	40 (10.1)
	6^th^	76 (19.1)
Do you have any underlying medical problems?	No	352 (88.7)
	Yes	45 (11.3)
	Anemia	9 (20)
	Asthma	18 (40)
	Asthma and GERD	1 (2.2)
	DM	4 (8.8)
	GERD, hiatal hernia, anemia	1 (2.2)
	GERD, hiatal hernia, disc prolapse	1 (2.2)
	HTN	1 (2.2)
	IBS	4 (8.8)
	Mental illness (bipolar)	1 (2.2)
	Nausea, shortness of breath, GERD	1 (2.2)

Of the participants, 18.4% had a history of smoking tobacco cigarettes, and 13.6% continued to smoke; of tobacco cigarette smokers, 70.5% smoked 10 or fewer cigarettes in the past seven days, and 55.7% were smokers for less than five years. A total of 27.5% smoked hookah (sheeshah) and 17.6% continued to smoke; of sheesha smokers, 65.9% were smokers for less than five years. A total of 35% were current E-cigarette smokers, while only 14.4% had quit; of the current E-cigarette users, 71.3% used E-cigarettes for four to seven days a week and 35% used them for more than two years, and 33% reported that one cartridge (defined as a 60 mL bottle with 3 mg of nicotine) lasts two to four weeks (Table 2). 

**Table 2:** Distribution of studied students according to their smoking data (cigarettes, hookah (sheeshah), electronic cigarettes (vape)) (n = 397)

Variable		n (%)
Do you smoke tobacco cigarettes?	No	324 (81.6)
	Yes	73 (18.4)
Do you have a history of smoking tobacco cigarettes?	Continue to smoke	54 (13.6)
	No history of smoking	231 (58.2)
	Quit	112 (28.2)
If you are a smoker,how many cigarettes a day did you smoke in the past seven days? (n = 54)	10 cigarettes or less	38 (70.5)
	11-20	12 (22.2)
	21-30	3 (5.5)
	31 or more	1 (1.8)
For how long have you smoked? (n = 54)	10-15 years	3 (5.5)
	5-10 years	19 (35.1)
	Less than 5 years	30 (55.7)
	More than 15 years	2 (3.7)
Do you use hookah (sheeshah) or not?	No	288 (72.5)
	Yes	109 (27.5)
Do you have a history of smoking hookah (sheeshah)?	Continue to smoke	70 (17.6)
	No history of smoking	219 (55.2)
	Quit	108 (27.2)
For how long have you smoked hookah (sheeshah)? (n = 70)	10-15 years	3 (4.2)
	5-10 years	15 (21.4)
	Less than 5 years	46 (65.9)
	More than 15 years	6 (8.5)
Do you, or have you at any point previously, used electronic cigarettes (vape)?	Current user	139 (35)
	Never	201 (50.6)
	Quit	57 (14.4)
How many days per week do you use electronic cigarettes (vape)? (n = 39)	1 day	12 (8.6)
	2-3 days	28 (20.1)
	4-7 days	99 (71.3)
For how long have you been using electronic cigarettes (vape)? (n = 139)	From one year to less than two years	37 (26.6)
	From six months to 1 year	33 (23.7)
	Less than six months	20 (14.3)
	More than two years	49 (35.4)
On average, how long does one cartridge (defined as a 60 mL bottle with 3 mg of nicotine) last with you? (n = 139)	0-2 weeks	31 (22.6)
	2-4 weeks	46 (33)
	4-6 weeks	28 (20.1)
	6-8 weeks	14 (10)
	More than 8 weeks	20 (14.3)

**Figure 1:**
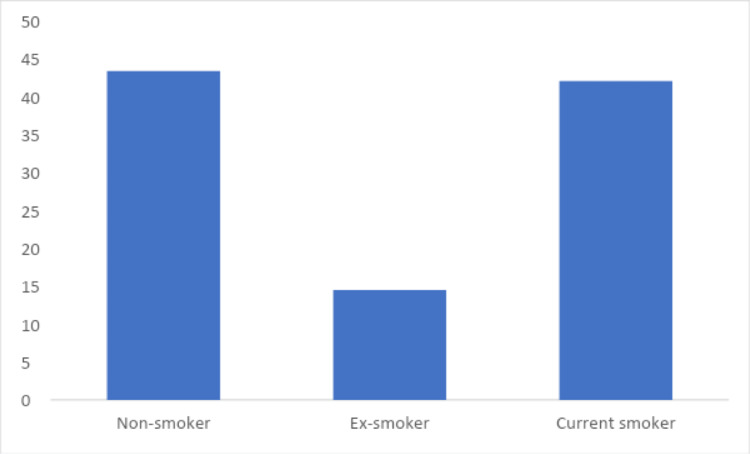
Percentage distribution of students according to prevalence of any type of smoking (cigarette, hookah or electronic cigarettes) (n = 397)

**Figure 2:**
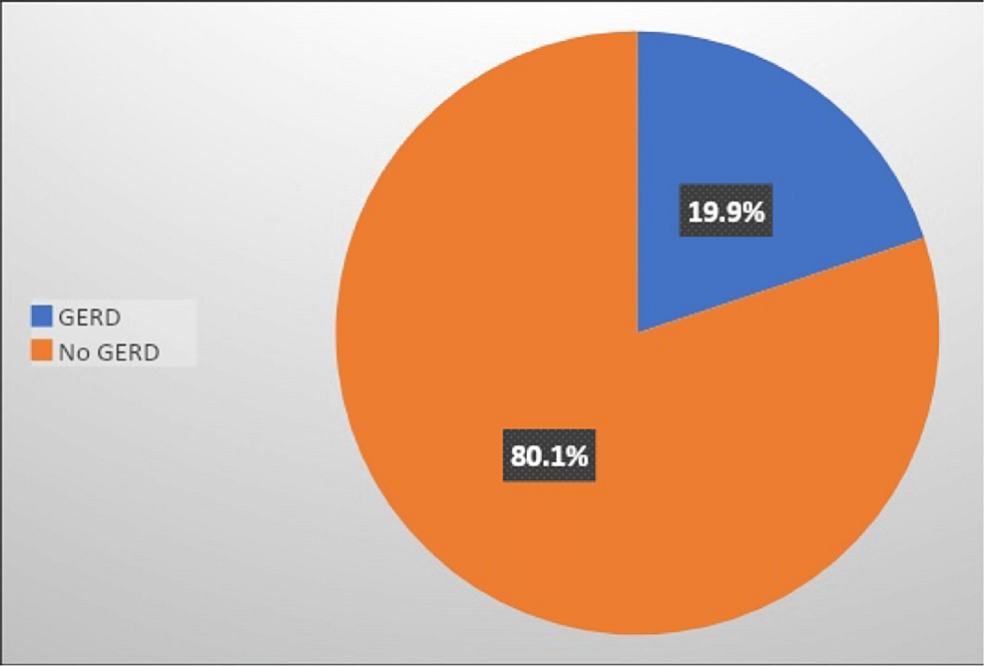
Percentage distribution of students according to GERD prevalence based on the GerdQ scores classification (n = 397) GERD, Gastroesophageal Reflux Disease; GerdQ, Gastroesophageal Reflux Disease Questionnaire

 Figure 1 illustrates that 43.1% of participating students currently smoked (cigarettes, sheeshah, or E-cigarettes). Based on the GerdQ scores classification, 19.9% of the studied students had GERD, as illustrated in Figure 2.

Females with no underlying medical problems or those who had asthma as a chronic disease had a significantly higher percentage of having GERD (p=<0.05) (Table 3). Moreover, male participants had a significantly higher mean BMI compared to females (p=<0.05) Table 4). At the same time, a non-significant relationship was found between BMI and smoking tobacco cigarettes, hookah, or E-cigarettes (p=>0.05).

**Table 3:** The association between GERD prevalence and participants' demographics, academic data, and underlying medical problems (No=397) * = Mann-Whitney test BMI, Body Mass Index; GERD, Gastroesophageal Reflux Disease; DM, Diabetes Mellites; HTN, Hypertension; IBS, Irritable Bowel Syndrome

Variable		GERD		χ2	p-value
		No, n (%)	Yes, n (%)		
Age	18-20	120 (37.7)	25 (31.6)	3.68	0.297
	21-23	145 (455.6)	36 (45.6)		
	24-26	44 (13.8)	17 (21.5)		
	Older than 26	9 (2.8)	1 (1.3)		
Gender	Female	212 (66.7)	63 (79.7)	5.08	0.024
	Male	106 (33.3)	16 (20.3)		
Marital status	Married	18 (5.7)	8 (10.1)	2.06	0.151
	single	800 (94.3)	71 (89.9)		
Do you have any underlying medical problems?	No	296 (93.1)	56 (70.9)	31.01	<0.001
	Yes	22 (6.9)	23 (29.1)		
If yes to above question, please specify (N=45)	Anemia	3 (0.9)	6 (7.6)	6.6	<0.001
	Asthma	9 (2.8)	9 (11.4)		
	DM	4 (1.3)	0 (0.0)		
	GERD	1 (0.3)	2 (2.5)		
	GERD and Asthma	1 (0.3)	0 (0.0)		
	GERD, hiatal hernia, anemia	0 (0.0)	1 (1.3)		
	GERD, hiatal hernia, disc prolapse	0 (0.0)	1 (1.3)		
	HTN	0 (0.0)	1 (1.3)		
	IBS	4 (1.3)	0 (0.0)		
	Mental illness (bipolar)	0 (0.0)	1 (1.3)		
	Nausea, shortness of breath, GERD	0 (0.0)	1 (1.3)		
	BMI	22.77 ± 4.88	22.79 ± 5.89	0.67*	0.05

**Table 4:** The association between BMI and participants' gender and smoking status (n = 397) BMI, Body Mass Index

Variable		BMI (mean ± SD)	Mann-Whitney test	p-value
Gender	Female	22.41 ± 4.94	1.99	0.046
	Male	23.6 ± 5.034		
Do you smoke tobacco cigarette?	No	22.63 ± 4.89	0.72	0.466
	Yes	23.42 ± 5.88		
Do you smoke hookah (sheeshah)?	No	22.49 ± 4.88	1.69	0.09
	Yes	23.53 ± 5.55		
Do you smoke electronic cigarettes (vape)?	No	22.71 ± 5.07	0.42	0.67
	Yes	22.9 ± 5.14		
Smoking tobacco cigarette	Current user	23.23 ± 5.47	2	0.27
	Never	22.43 ± 5		
	Quit	23.27 ± 5.07		
Smoking hookah (sheeshah)	Current user	23.74 ± 5.75	2	0.098
	Never	22.31 ± 4.96		
	Quit	23.09 ± 4.83		
Smoking electronic cigarettes (vape)	Current user	22.9 ± 5.14	2	0.871
	Never	22.6 ± 4.95		
	Quit	23.1 ± 5.52		

**Figure 3:**
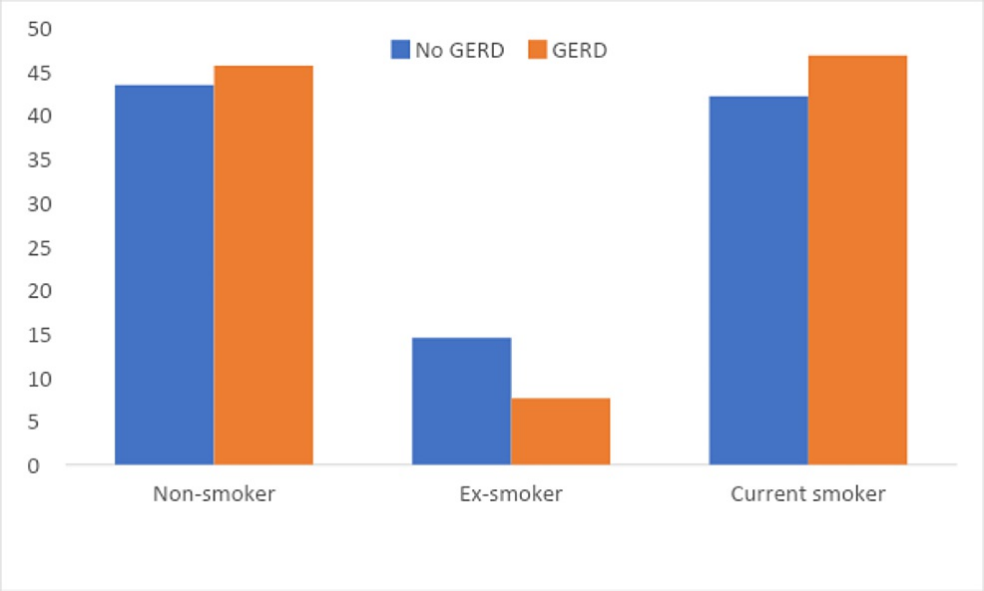
Relationship between GERD prevalence and prevalence of type smoking (cigarette, hookah, or E-cigarettes) (n = 397) GERD, Gastroesophageal Reflux Disease.

On the other hand, there was no significant relationship between GERD prevalence and smoking status (cigarettes, hookah, and E-cigarettes) (p=>0.05) (Table 5). At the same time, there was no significant relationship found between GERD prevalence and the prevalence of any smoking method (cigarette, hookah, or E-cigarettes) (p=>0.05) (Figure 3) (Table 6).

**Table 5:** Relationship between GERD prevalence and participants' smoking data (cigarettes, hookah (sheeshah), electronic cigarettes (vape)) (n = 397) GERD, Gastroesophageal Reflux Disease

Variable		GERD			
		No, n (%)	Yes, n (%)		
Do you smoke tobacco cigarette?	No	264 (83)	60 (75.9)	2.1	0.147
	Yes	54 (17)	19 (24.1)		
Do you have history of smoking tobacco cigarettes?	Continue to smoke	40 (12.6)	14 (17.7)	1.42	0.49
	No history of smoking	187 (58.8)	44 (55.7)		
	Quit	91 (28.6)	21 (26.6)		
If you are a smoker, how many cigarettes a day did you smoke in the past seven days? (n = 54)	10 cigarettes or less	26 (8.2)	12 (15.2)	4.05	0.283
	11-20	11 (3.5)	1 (1.3)		
	21-30	2 (0.6)	1 (1.3)		
	31 or more	1 (0.3)	0 (0.0)		
For how long have you smoked? (n= 54)	10-15 years	3 (0.9)	0 (0.0)	4.01	0.404
	5-10 years	15 (4.7)	4 (5.1)		
	Less than 5 years	21 (6.6)	9 (11.4)		
	More than 15 years	1 (0.3)	1 (1.3)		
Do you use hookah (sheeshah)?	No	232 (72)	56 (70.9)	0.13	0.712
	Yes	86 (27)	23 (29.1)		
Do you have history of smoking hookah (sheeshah)?	Continue to smoke	56 (17.6)	14 (17.7)	0.02	0.988
	No history of smoking	176 (55.3)	43 (54.4)		
	Quit	86 (27)	22 (27.8)		
For how long have you smoked hookah (sheeshah)? (n = 70)	10-15 years	2 (0.6)	1 (1.3)	2.26	0.687
	5-10 years	11 (3.5)	4 (5.1)		
	Less than 5 years	37 (11.6)	9 (11.4)		
	More than 15 years	6 (1.9)	0 (0.0)		
Do you, or have you at any point previously, used electronic cigarettes (vape)?	Current user	109 (34.3)	30 (38)	0.47	0.788
	Never	162 (50.9)	39 (49.4)		
	Quit	47 (14.8)	10 (12.7)		
How many days per week you use electronic cigarettes (vape)? (n = 139)	1 day	10 (3.1)	2 (2.5)	0.58	0.899
	2-3 days	22 (6.9)	6 (7.6)		
	4-7 days	77 (24.2)	22 (27.8)		
For how long have you been using electronic cigarettes (vape)? (n = 139)	From one year to less than two years	27 (8.5)	10 (12.7)	3.74	0.442
	From six months to 1 year	24 (7.5)	9 (11.4)		
	Less than six months	18 (5.7)	2 (2.5)		
	More than two years	40 (12.6)	9 (11.4)		
On average, how long does one cartridge (defined as a 60 mL bottle with 3 mg of nicotine) last with you? (n = 139)	0-2 weeks	26 (8.2)	5 (6.3)	3.46	0.629
	2-4 weeks	33 (10.4)	13 (16.5)		
	4-6 weeks	23 (7.2)	5 (6.3)		
	6-8 weeks	10 (3.1)	4 (5.1)		
	More than 8 weeks	17 (5.3)	3 (3.8)		

**Table 6:** Relationship between GERD prevalence and prevalence of type smoking (cigarette, hookah, or electronic cigarettes) (n = 397) GERD, Gastroesophageal Reflux Disease

	No GERD (%)	GERD (%)
Non-smoker	43.4	45.6
Ex-smoker	14.5	7.6
Current smoker	42.1	46.8

## Discussion

In this cross-sectional study, we assessed the prevalence of GERD and its relation with E-cigarette smoking among 397 students at Jeddah universities using a well-validated GERD score (GerdQ).

E-cigarette has been promoted as a risk-free substitute for smokers of conventional cigarettes. However, recent research has demonstrated that the risks associated with using E-cigarettes can be comparable to those associated with tobacco use [15].

Our study revealed that E-cigarettes have become popular among university students. The prevalence of E-cigarette smoking is 35%, significantly higher than smoking hookah (17.6%) and tobacco cigarette smoking (13.6%). A similar percentage was reported from three different health science schools (College of Medicine, College of Applied Medical Science, College of Nursing, and College of Pharmacy) in the Jeddah-western region of Saudi Arabia, showing that E-cigarette use was 27.7%, almost twice as much as conventional cigarette smokers (14.1%) [11]. Another study conducted among health professional students in the United States demonstrated that E-cigarettes increased in prevalence [16]. The reason for the increasing current prevalence of E-cigarettes can be curiosity to try new, practical products.

Compared to traditional smoking, E-cigarette smoking is associated with a higher incidence of GERD among Jeddah college students who participated in the current study. In addition to having GERD, 38% of the students use E-cigarettes. This could be conflicted with stress, which is also one of the risk factors of GERD. Smoking is the primary calming agent among people, and college students frequently deal with challenging situations that could be stressful. Our study shows that 43.1% of college students are smokers and 13.1% are ex-smokers, while another study showed 35.3% of college students smoke [17]. This finding could be due to the perceived stress scale, coping inventory for stressful situations, emotion-oriented coping, task-oriented coping, or avoidance-oriented coping [18]. 

In addition, one-third of our smokers had GERD, and three cross-sectional studies have indicated a significant positive association between GERD symptoms and smoking [19-21]. The potential effects of chronic smoking could explain the significant positive association between GERD symptoms and smoking. These effects include smoking stimulating gastric acid secretion and decreasing the lower esophageal sphincter pressure. Moreover, smoking impairs the healing process of the lower esophageal inflammation resulting from reflux [22,23]. This study has shown that the number of participants who were previously smokers and have GERD symptoms is less than participants that have no GERD symptoms. In another study that was done in Norway, it was seen that participants who are overweight use anti-reflex medications for less than one week and those who have minor GERD symptoms had no improvement in their GERD symptoms despite cessation of smoking [24].

In the current study, GERD was more common in females than males. This difference may be due to hormonal factors. Also, we found that E-cigarette is associated with high BMI in male individuals. Another study shows that E-cigarettes are associated with a lower BMI [25].

The treatment of GERD involves lifestyle changes, such as weight loss, raising the head of the bed when resting, and avoiding eating for a minimum of three hours before sleeping. Apart from lifestyle modifications, GERD is frequently managed with pharmacological interventions (proton pump inhibitors and histamine type 2 (H2) receptor blockers). To restore normal gastrointestinal tract function, mechanical repair might be required through endoscopy (transoral incisionless fundoplication (TIF) and Stretta®) or surgery. Currently, laparoscopic Nissen fundoplication (LNF) is the recognized standard of care for patients with GERD that has not responded to other treatments [26].

To the best of our knowledge, a few types of research were done to assess the relationship between GERD and E-cigarettes in Saudi Arabia. Our study has limitations, including the inclusion criteria, mainly focusing on university students in Jeddah. Therefore, further studies should be done to include a broader population. Also, the study did not specify the type of E-cigarettes. Another limitation was the paucity in the literature.

## Conclusions

In this study, we concluded that there is an increase in the prevalence of E-cigarette smoking among college students in Jeddah compared to regular smoking and hookah. However, we did not find a significant association between E-cigarettes and GERD. High BMI is a confounding factor for the predisposition of GERD in E-cigarettes smoking men.

Finally, we recommend health professional education for all university students about the risks and hazards of E-cigarettes. Other studies are required to conclude more side effects of E-cigarettes due to their widespread among young adults.
